# Diagnostic Discordance in Recurrent Pregnancy Loss: Hysteroscopy Resolves Ultrasound–MRI Disagreement in Septate Uterus, but Concurrent Ovulation Induction Precludes Causal Attribution of the Reproductive Outcome

**DOI:** 10.1002/ccr3.73515

**Published:** 2026-09-23

**Authors:** Iftekhar Ahmed Sakib, Ayesa Perveen, Nishad Tasnim

**Affiliations:** ^1^ Department of Obstetrics and Gynecology Shaheed Suhrawardy Medical College Dhaka Bangladesh; ^2^ University of Dhaka Dhaka Bangladesh

**Keywords:** diagnostic discordance, hysteroscopy, recurrent pregnancy loss, septate uterus, septoplasty

## Abstract

Congenital uterine anomalies are an important and potentially treatable contributor to recurrent pregnancy loss (RPL). The septate uterus is the commonest Müllerian anomaly linked to first‐trimester loss, and its differentiation from the benign arcuate uterus is essential for management, yet imaging modalities may disagree. We report the case of a 23‐year‐old woman (G2P0A2) who presented with oligomenorrhea and two consecutive spontaneous first‐trimester miscarriages. Initial transvaginal ultrasound suggested a septate uterus, whereas pelvic magnetic resonance imaging indicated an arcuate uterus, resulting in diagnostic confusion. Diagnostic hysteroscopy revealed a thick, fibrous partial septum, and simultaneous hysteroscopic septoplasty was performed. A complete RPL evaluation was not possible: antiphospholipid antibody testing, parental karyotyping, and genetic analysis of products of conception were unavailable, so alternative etiologies could not be excluded and the septum cannot be established as the sole cause of the miscarriages. Comprehensive endocrine assessment was likewise unavailable, leaving ovulatory dysfunction incompletely characterized; a lean polycystic ovary syndrome phenotype could not be excluded. Following septoplasty and subsequent ovulation induction with letrozole, she conceived and delivered a healthy male infant at term by cesarean section. Because conception occurred only after letrozole, the relative contributions of these two sequential interventions cannot be distinguished. Second‐look hysteroscopy was not performed, so restoration of a normal cavity was never objectively confirmed and residual septum or intrauterine adhesions could not be excluded before conception. This case demonstrates the diagnostic value of hysteroscopy in resolving discordant imaging while emphasizing that the outcome must not be attributed to septoplasty alone.

## Key Clinical Message

When transvaginal ultrasound and MRI disagree in recurrent pregnancy loss, hysteroscopy definitively clarifies intracavitary anatomy and permits same‐setting treatment. Its value here is diagnostic, not therapeutic: because septoplasty was followed by ovulation induction, causality cannot be attributed to either, and without second‐look hysteroscopy anatomical success remains unconfirmed.

**Abbreviations**
ASRM: American Society for Reproductive Medicine
BMI: body mass index
ESGE: European Society for Gynecological Endoscopy
ESHRE: European Society of Human Reproduction and Embryology
LSCS: lower segment cesarean section
MRI: magnetic resonance imaging
RPL: recurrent pregnancy loss
T2‐WI: T2‐weighted imaging
TVS: transvaginal sonography

## 1 Introduction

Recurrent pregnancy loss (RPL), which affects 1%–2% of couples during their reproductive years, is broadly defined as the loss of two or more clinically confirmed pregnancies before 20–24 weeks of gestation [1]. RPL has multifactorial etiologies, including genetic, anatomical, endocrine, immunologic, and thrombophilic causes. Congenital uterine defects are well‐established anatomical causes of RPL, with the septate uterus being the most common and closely linked to first‐trimester loss [2]. A septate uterus is defined as a congenital uterine anomaly characterized by a fibromuscular septum dividing the endometrial cavity while maintaining a normal or slightly concave exterior fundal shape, resulting from incomplete resorption of the Müllerian ducts. An arcuate uterus is considered a mild anatomical variation with little clinical importance, as it presents with a shallow fundal indentation and minimal distortion of the uterine cavity. It is important to distinguish between these two abnormalities because an arcuate uterus is often treated expectantly, whereas a septate uterus can be surgically repaired. Non‐invasive imaging with MRI and transvaginal sonography (TVS) may nevertheless yield discordant findings, particularly near diagnostic thresholds, delaying appropriate treatment [3]. Hysteroscopy remains the gold standard for diagnosing intrauterine abnormalities because it allows direct visualization of the uterine cavity and simultaneous therapeutic intervention. It is complementary to imaging that assesses the external uterine contour [4]. This case report illustrates how hysteroscopy clarified the diagnosis when discordance existed between imaging modalities and highlights the importance of addressing coexisting reproductive factors in order to achieve a successful pregnancy outcome.

## 2 Case Presentation

### 2.1 Patient Information

A 23‐year‐old Bangladeshi woman, Gravida 2, Para 0, Abortus 2 (G2P0A2), was referred after two consecutive spontaneous first‐trimester miscarriages at 12 and 8 weeks. Both conceptions were achieved naturally without assistance. Her medical history was otherwise unremarkable, with no prior uterine surgeries, pelvic inflammatory disease, or diagnosed endocrinopathies. She had no personal or family history of thrombophilia, autoimmune disorders, or known genetic conditions. Standard RPL evaluation was undertaken in accordance with locally available protocols and international recommendations. The patient underwent thyroid function testing (TSH), serum prolactin measurement, glycated hemoglobin (HbA1c), and detailed pelvic imaging. Given her history of two first‐trimester losses without suggestive clinical features (no history of thrombosis, autoimmune disease, or fetal structural anomalies), antiphospholipid antibody testing, inherited thrombophilia screening, and parental karyotyping were not performed. These investigations were deferred due to both limited resource availability and the absence of clinical indicators that would increase pretest probability. Products of conception from prior miscarriages were not available for genetic analysis.

### 2.2 Clinical Findings

The patient's chief complaint was her history of recurrent miscarriage, accompanied by concerns regarding future fertility. She reported long‐standing oligomenorrhea, with spontaneous menstrual cycles occurring at intervals of 8–10 weeks, but denied dysmenorrhea or intermenstrual bleeding. A general physical examination was within normal limits; her body mass index (BMI) was 20 kg/m^2^. Bimanual examination revealed an anteverted, mobile uterus of normal size with no adnexal masses or tenderness.

## 3 Methods

### 3.1 Differential Diagnosis

The patient's oligomenorrhea (cycles every 8–10 weeks) was evaluated for underlying causes of ovulatory dysfunction.

The primary conditions considered included Polycystic Ovary Syndrome, Functional Hypothalamic Amenorrhea, Thyroid Disease, and Hyperprolactinemia. Initial evaluation included measurement of thyroid‐stimulating hormone (TSH), serum prolactin, and glycated hemoglobin (HbA1c), all of which were within normal limits, thereby excluding overt thyroid dysfunction, hyperprolactinemia, and significant metabolic derangement.

Given the patient's normal body mass index (20 kg/m^2^) and absence of clinical features of hyperandrogenism (e.g., hirsutism, acne), classic phenotypes of polycystic ovary syndrome were considered less likely. Functional hypothalamic amenorrhea was considered less probable in the absence of identifiable contributing factors such as significant weight loss, excessive physical activity, or psychosocial stress.

The endocrine evaluation of this patient's oligomenorrhea was incomplete, and the etiology of her ovulatory dysfunction consequently remains unresolved. Serum luteinizing hormone (LH) and follicle‐stimulating hormone (FSH) with the LH:FSH ratio, total and free testosterone, dehydroepiandrosterone sulfate, androstenedione, sex hormone‐binding globulin with the free androgen index, 17‐hydroxyprogesterone (to exclude non‐classic congenital adrenal hyperplasia), anti‐Müllerian hormone, and mid‐luteal progesterone were not measured, and dedicated ultrasonographic assessment of ovarian morphology (antral follicle count and ovarian volume) was not undertaken. The Rotterdam criteria for polycystic ovary syndrome could therefore not be formally applied, and a lean or non‐classic PCOS phenotype, mild functional hypothalamic dysfunction, and early diminished ovarian reserve all remain plausible but unproven explanations for cycles occurring every 8–10 weeks.

We regard this as a substantive limitation rather than a technical omission. Because ovulatory dysfunction was the indication for letrozole, and because conception followed only after ovulation induction, an unresolved endocrine diagnosis leaves open the possibility that oligo‐anovulation, rather than the uterine septum, was the dominant contributor to this patient's reproductive history. The interpretation of the eventual outcome is therefore constrained at its root by an incompletely characterized ovulatory disorder. Women with recurrent pregnancy loss and oligomenorrhea should therefore undergo a full gonadotropin and androgen profile with ovarian morphological assessment alongside surgical management.

This framework informed the decision to add ovulation induction to surgical management.

### 3.2 Further Investigations

The primary diagnostic focus was identifying a potential anatomical cause for RPL. TVS suggested a septate uterus, whereas MRI suggested an arcuate uterus.

### 3.3 Transvaginal Sonography (TVS)

**FIGURE 1:**
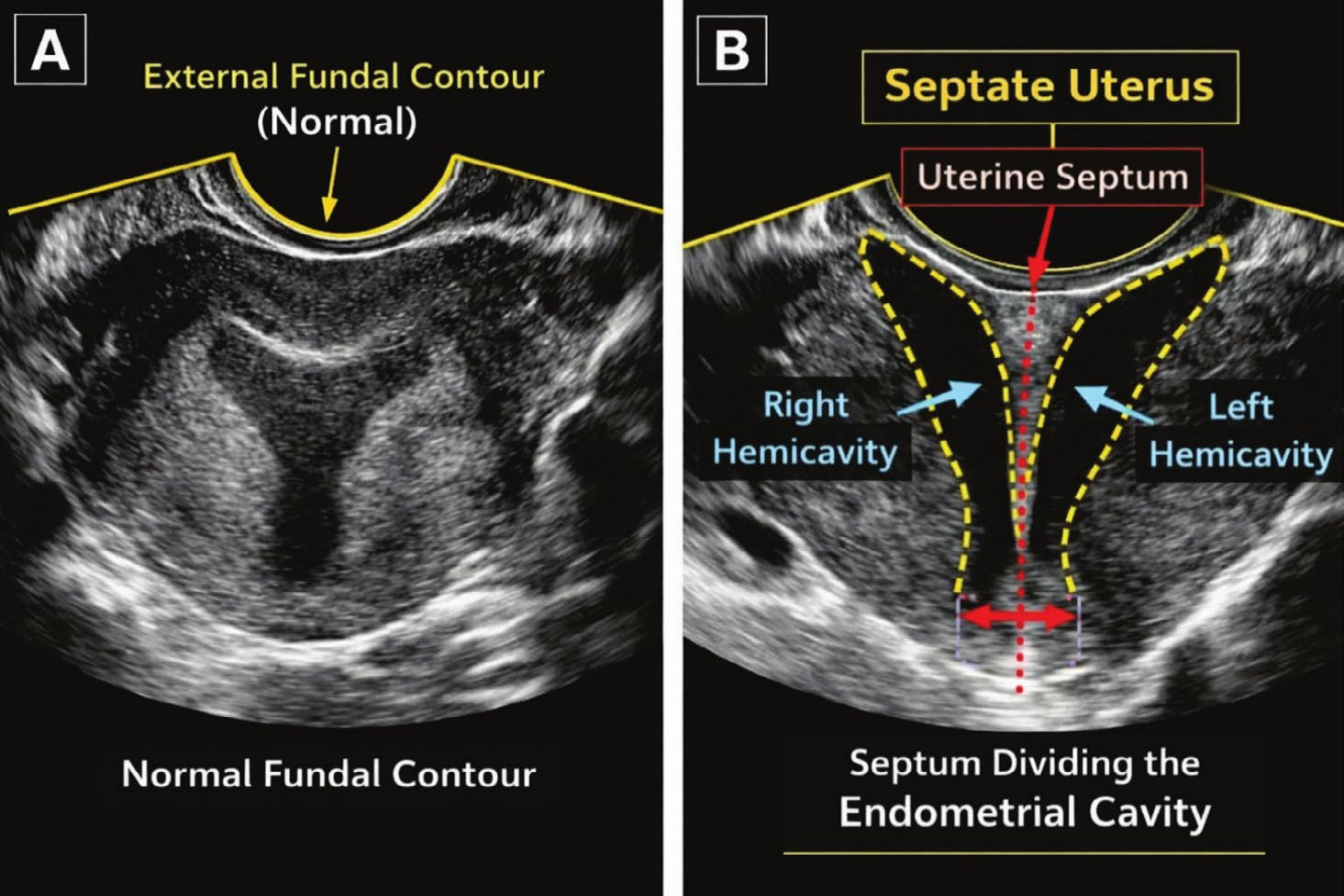
Transvaginal ultrasound demonstrating a septate uterus. (A) Transvaginal ultrasound image in the coronal plane showing the uterine cavity with a preserved, smooth external fundal contour and no fundal cleft, indicating a normally contoured uterine fundus. (B) Midline echogenic intrauterine septum (solid arrows) extending inferiorly from the fundus and dividing the endometrial cavity into two symmetric hemicavities (dashed lines). The intact external contour without fundal indentation supports a septate rather than bicornuate uterus (ASRM 2021 criteria).

Performed in the mid‐follicular phase, TVS depicted an anteverted uterus with normal myometrial echotexture and a distinct fundal indentation dividing the endometrial lining into two echoes of 1.11 cm and 0.81 cm, converging above the internal cervical os. The external contour was convex and smooth. The impression was a septate uterus (Figure 1A,B).

### 3.4 Pelvic Magnetic Resonance Imaging (MRI)

**FIGURE 2:**
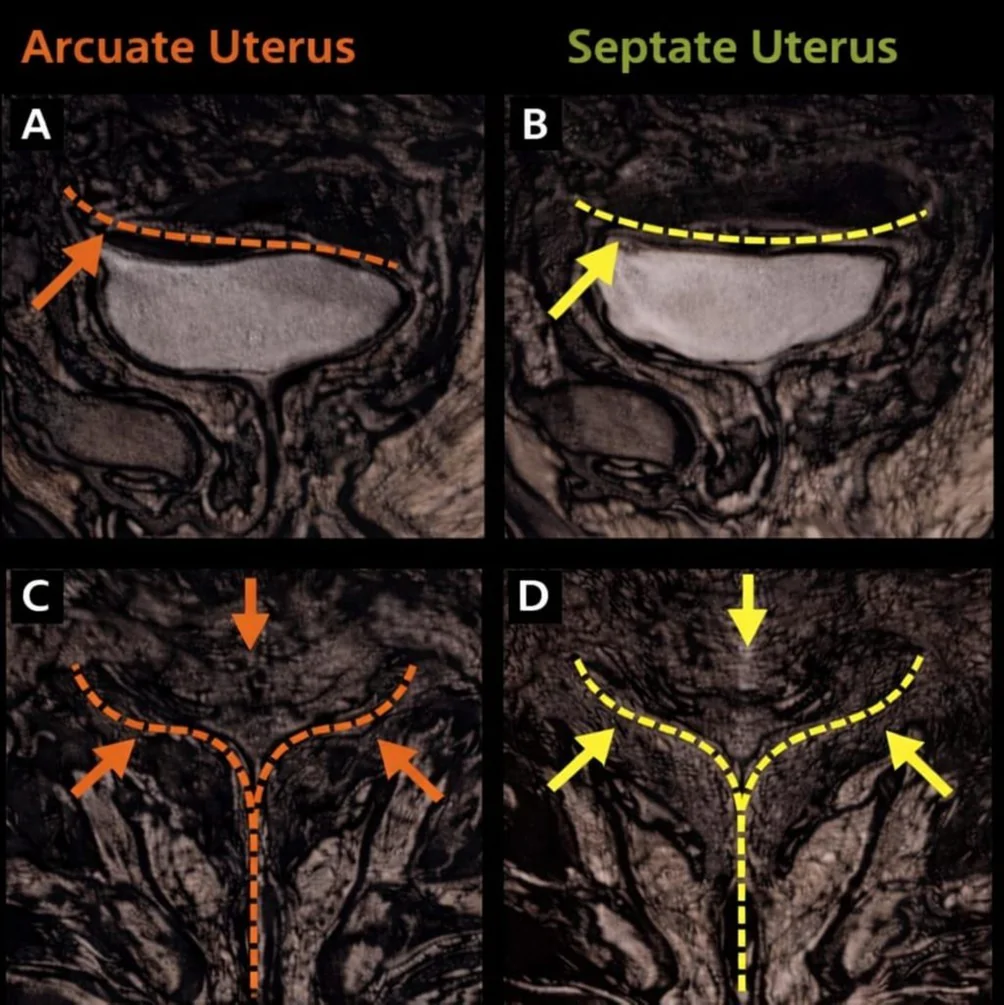
Pelvic MRI illustrates diagnostic discordance between arcuate and septate uterus. (A) Sagittal T2‐weighted MRI: Shallow concave indentation of the endometrial fundus (orange dashed line and arrow), consistent with an arcuate uterus. (B) Sagittal T2‐weighted MRI: Linear midline indentation extending inferiorly toward the cavity (yellow dashed line and arrow), consistent with a septate uterus. (C) Coronal T2‐weighted MRI: Broad, shallow fundal indentation without extension into the cavity (orange dashed line and arrows), characteristic of an arcuate uterus. (D) Coronal T2‐weighted MRI: Well‐defined midline fibrous septum extending toward the cavity (yellow dashed line and arrows), characteristic of a septate uterus.

Obtained for definitive characterization, MRI (T2‐weighted sequences) confirmed an anteverted uterus. It demonstrated a mild fundal myometrial indentation measuring approximately 1.8 cm in depth. Critically, the external fundal contour remained convex, with no evidence of a sulcus. The radiological impression was an arcuate uterus (Figure 2A–D).

This contradiction between TVS (a clinically significant anomaly) and MRI (a normal variant) created the core diagnostic challenge.

### 3.5 Definitive Diagnostic Modality—Hysteroscopy

**FIGURE 3:**
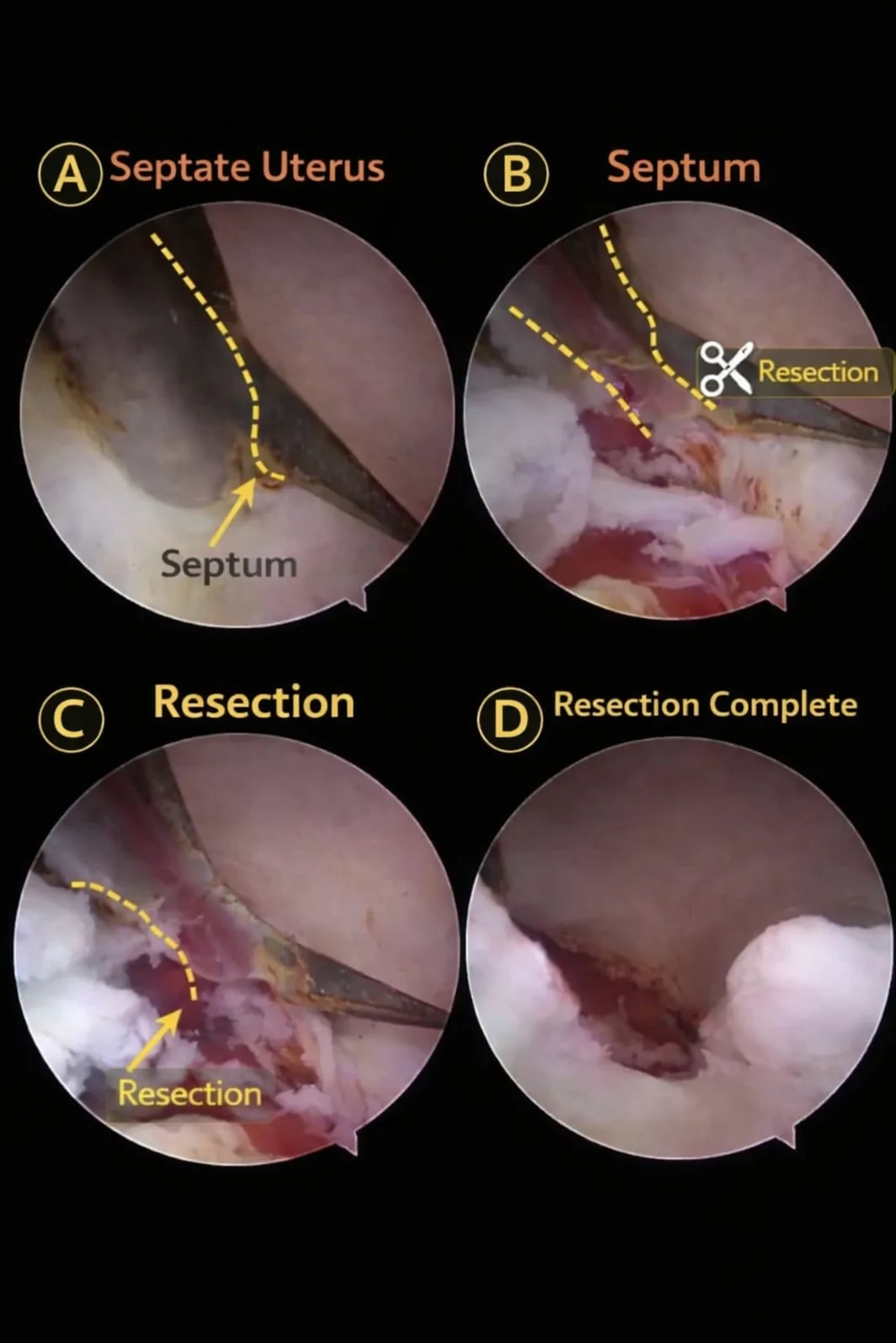
Hysteroscopic resection of a uterine septum using scissors. Representative hysteroscopic images demonstrating (A) the septum as a fibrous midline structure dividing the cavity; (B) placement of hysteroscopic scissors at its base; (C) progressive resection under controlled scissor dissection; and (D) post‐resection restoration of a single uterine cavity.

Hysteroscopy performed on December 7, 2022 with a 2.9 mm 30‐degree rigid hysteroscope with a continuous‐flow operating sheath via a vaginoscopic approach (no speculum or tenaculum used). Then after entry into the uterine cavity, a thick fibrous septum was identified extending approximately 2.5 cm into the uterine cavity, ending well above the internal os. Both tubal ostia were clearly visualized, confirming the diagnosis of a partial septate uterus (Figure 3A–D). The full operative sequence is shown in (Video 1).

**VIDEO 1:** Hysteroscopic resection of the uterine septum (still frame extracted from this video). The video records the operative sequence illustrated in Figure 3: Vaginoscopic entry into the uterine cavity with a 2.9 mm 30‐degree rigid hysteroscope, identification of the fibrous midline septum with both tubal ostia in view, progressive midline incision with 5‐Fr hysteroscopic scissors, and the post‐resection appearance of a single symmetrical cavity. Duration: 4 min 19 s. File format: MP4 (MPEG‐4/QuickTime container) with H.264 video, 480 × 848 pixels, 29.6 frames per second, file size 46 MB. The submitted file carries no audio track. Written informed consent for publication of the intraoperative recording was obtained from the patient, and no identifying information appears in any frame. Video content can be viewed at https://onlinelibrary.wiley.com/doi/10.1002/ccr3.73515.

### 3.6 Treatment

Given the history of RPL and the ambiguous imaging, diagnostic hysteroscopy was undertaken with provision for concurrent operative intervention, under total intravenous anesthesia. Hysteroscopic inspection confirmed the diagnosis. 5‐Fr hysteroscopic scissors were introduced through the continuous‐flow operative sheath for septal resection. The septum was meticulously incised in a midline, progressive fashion until a single, symmetrical cavity was restored, with both tubal ostia in a single field of vision. Hemostasis was maintained. The procedure was completed without complication.

A second‐look hysteroscopy was not performed because of resource limitations and the absence of postoperative symptoms such as abnormal bleeding or suspected intrauterine adhesions. Where resources permit, cavity reassessment 4–6 weeks after septoplasty, by second‐look hysteroscopy or, if unavailable, three‐dimensional or saline‐infusion sonography, should precede attempts at conception. We wish to state explicitly that, in the absence of second‐look hysteroscopy, the anatomical success of this procedure cannot be confirmed. Restoration of a single symmetrical cavity was documented only by the operator's intraoperative view at the conclusion of resection; no objective reassessment of the cavity was obtained at any point before conception. It therefore cannot be established that the patient entered her subsequent pregnancy with a normal uterine cavity: residual septal tissue, not infrequently identified after an apparently complete resection, or de novo intrauterine adhesions may have been present and undetected. Any inference that the subsequent term pregnancy followed complete anatomical correction is, for this reason, unverifiable.

### 3.7 Follow‐Up and Outcomes

The patient's postoperative course was unremarkable. In light of her persistent oligomenorrhea, ovulation induction was initiated with letrozole 5 mg. She conceived spontaneously in her second treatment cycle. The antenatal period was uneventful. At 38 weeks and 4 days of gestation, a lower segment cesarean section was performed for breech presentation. She delivered a healthy male infant with a birth weight of 2.8 kg and Apgar scores of 9 and 10 at one and 5 min, respectively. At her most recent postpartum visit, both mother and infant were thriving. A chronological summary of key clinical events, diagnostic steps, and therapeutic interventions is provided in Table 1.

**TABLE 1:** Summary of clinical findings, diagnostic workup and management timeline.

Timeline for the clinical events		
Date	Clinical event	Significance
2021–2022	Two first‐trimester miscarriages	Trigger for RPL evaluation
22 Sep 2022	Initial consultation and TVS	First suspicion of septate uterus
5 Dec 2022	Pelvic MRI	Discordant diagnosis: arcuate uterus
7 Dec 2022	Diagnostic and operative hysteroscopy	Definitive diagnosis and septoplasty
May 2023	Ovulation induction initiated	Management of oligo‐ovulation
Jul 2023	Conception confirmed	Successful pregnancy
3 Mar 2024	Term delivery by cesarean section	Favorable outcome
Present	Postpartum follow‐up	Planning future pregnancy

## 4 Discussion

**FIGURE 4:**
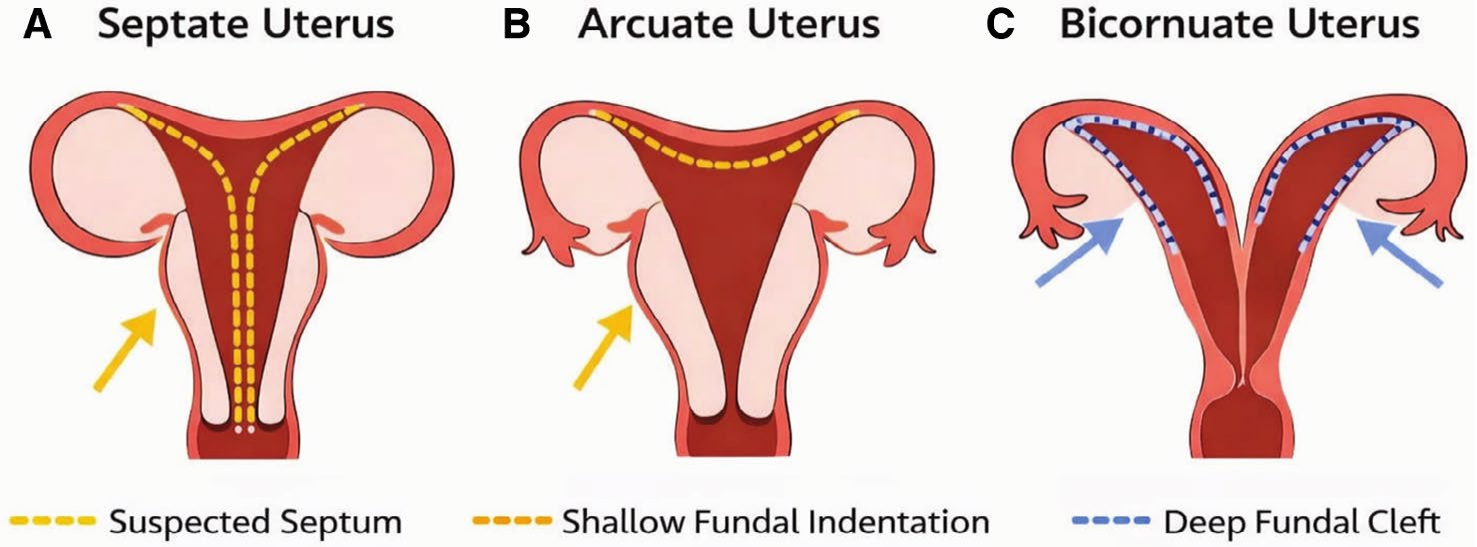
Schematic classification of Müllerian uterine anomalies (original author‐generated illustrations). (Panel A) Septate uterus, showing a central fibrous or fibromuscular septum extending from the fundus toward the endometrial cavity (yellow dashed line and arrow), with a smooth, convex external fundal contour. (Panel B) Arcuate uterus, showing a shallow, broad concave indentation of the endometrial fundus (orange dashed line and arrow) without extension into the uterine cavity. (Panel C) Bicornuate uterus, characterized by a deep external fundal cleft with separation of the uterine horns (blue dashed line and arrows). Symbols: Dashed yellow, orange, and blue lines with corresponding arrows delineate the septum, the shallow fundal indentation, and the deep fundal cleft, respectively. The same scheme is used in Figure 5. Image credit: These diagrams were created de novo by the authors in Adobe Illustrator with reference to ASRM classification descriptions; no published figure was traced or reproduced, and no permission was required. This figure contains schematic illustrations only and no radiological images.

**FIGURE 5:**
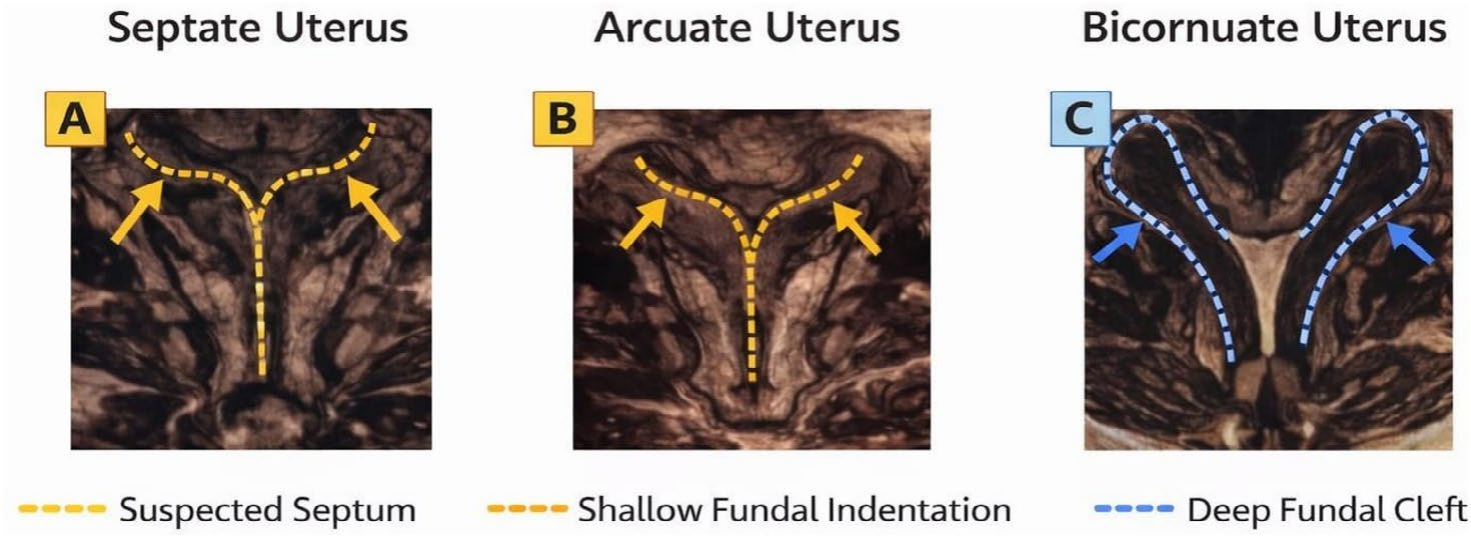
Representative T2‐weighted MRI appearances of septate, arcuate, and bicornuate uteri (educational images; NOT from the index patient). (Panel A) Coronal T2‐weighted MRI of a septate uterus, showing a linear midline indentation extending toward the endometrial cavity (yellow dashed line and arrows). (Panel B) Coronal T2‐weighted MRI of an arcuate uterus, showing a shallow concave indentation of the endometrial fundus (orange dashed line and arrows) without extension into the cavity. (Panel C) Coronal T2‐weighted MRI of a bicornuate uterus, showing a deep fundal cleft with divergent uterine horns (blue dashed line and arrows). Important note on provenance: (Panels A–C) are educational examples and do NOT correspond to the index patient, whose MRI is shown separately in Figure 2. They were obtained from copyright‐free/open‐access resources permitting reuse for non‐commercial scientific publication, with attribution per the source license.

This demonstration emphasizes the difficulties in detecting congenital uterine anomalies, particularly distinguishing between an arcuate and septate uterus using non‐invasive imaging techniques. The TVS and MRI disparity in this patient is a known disadvantage, especially when morphological measures are near diagnostic cutoffs [3, 5]. This reflects the known morphological overlap between septate, arcuate, and bicornuate uteri on imaging (Figures 4 and 5).

The American Society for Reproductive Medicine (ASRM) classifies an arcuate uterus as having a shallow indentation without a true septum, while a septate uterus is characterized by a normal or minimally indented external fundal contour with a significant internal fundal indentation [2]. Small variations in imaging technique, plane selection, or interpretation may lead to different classifications, and these standards are difficult to apply uniformly across modalities in incomplete septa. In this case, hysteroscopy demonstrated a fibrous intrauterine septum, making it the gold standard for diagnosis.

In addition to diagnosis, hysteroscopy provides the benefit of prompt therapeutic intervention. Hysteroscopic septoplasty is minimally invasive with low complication rates, and meta‐analyses report improved live birth and reduced miscarriage rates in women with septate uteri and poor reproductive history [6, 7].

Hysteroscopy remains an important tool for evaluation of intrauterine pathology, particularly when imaging findings are inconclusive. However, MRI and three‐dimensional ultrasonography remain valuable complementary modalities because they provide assessment of external fundal morphology and facilitate differentiation between septate and bicornuate uterus. The present case should therefore be regarded as an illustration of a successful multidisciplinary management pathway rather than evidence of a direct causal relationship between septoplasty and live birth.

Three‐dimensional ultrasonography has demonstrated strong diagnostic accuracy for Müllerian anomalies and might have reduced the initial ambiguity, but its unavailability here reflects practical limitations in many settings, illustrating the usefulness of hysteroscopy when imaging is unclear [5, 8].

A key limitation of this case is the absence of a complete RPL workup as recommended by ESHRE guidelines, including antiphospholipid antibody testing, parental karyotyping, and genetic analysis of products of conception. These were deferred because of limited resources and low clinical suspicion, so alternative etiologies cannot be excluded. Accordingly, the conclusion that the uterine septum caused the previous miscarriages remains speculative; the septum may have been a contributing factor or an incidental finding rather than the sole cause. This reservation is essential when interpreting the favorable reproductive outcome. Another limitation is the absence of second‐look hysteroscopy following septoplasty. We emphasize that this is a serious methodological limitation rather than a minor one. Without postoperative cavity reassessment it cannot be confirmed that no residual septum and no intrauterine adhesions were present before conception, so the anatomical success of the septoplasty remains unverified. This uncertainty compounds the impossibility of attributing the live birth to septum resection: neither the completeness of the anatomical correction nor its causal contribution to the outcome can be demonstrated from the data available in this case.

The successful term pregnancy followed two sequential interventions: hysteroscopic septoplasty and ovulation induction with letrozole. Given the patient's oligomenorrhea with cycles every 8–10 weeks, ovulatory dysfunction represented an independent reproductive factor that may have contributed to subfertility or pregnancy loss. Because conception occurred only after letrozole treatment, the contribution of septoplasty cannot be distinguished from that of ovulation induction, and the outcome must not be attributed to septum resection alone.

Recent literature has questioned the magnitude of reproductive benefit from septoplasty: the TRUST randomized controlled trial found no statistically significant improvement in live birth rates after septum resection compared with expectant management [9], although its methodology and selection criteria have been debated.

## 5 Conclusion

This case demonstrates the clinical value of hysteroscopy in clarifying intracavitary anatomy when non‐invasive imaging modalities provide discordant findings. Because genetic, immunologic, and endocrine causes were not completely excluded, and conception occurred only after ovulation induction, the favorable outcome observed here should not be attributed solely to septoplasty. Rather, it illustrates how integrated surgical and medical management may contribute to successful outcomes in selected patients. Equally, in the absence of second‐look hysteroscopy, anatomical success cannot be assumed. The principal contribution of this report is therefore diagnostic, demonstrating the role of hysteroscopy in resolving imaging discordance, rather than therapeutic.

## Author Contributions

**Iftekhar Ahmed Sakib:** conceptualization, data curation, formal analysis, investigation, methodology, project administration, resources, software, supervision, validation, visualization, writing – original draft, writing – review and editing. **Ayesa Perveen:** conceptualization, data curation, formal analysis, investigation, methodology, project administration, resources, supervision, validation, visualization, writing – review and editing. **Nishad Tasnim:** data curation, formal analysis, investigation, methodology, project administration, validation, visualization, writing – original draft, writing – review and editing.

## Funding

The authors have nothing to report.

## Consent

Written informed consent was obtained from the patient for publication of this case report and accompanying images. The consent form was reviewed and updated to ensure consistency with the final manuscript title, and all identifying and descriptive elements correspond to the present submission. The consent form is available for review by the journal editor upon request.

## Conflicts of Interest

The authors declare no conflicts of interest.

## Data Availability

The data that support the findings of this study are available from the corresponding author upon reasonable request. The data are not publicly available due to privacy and ethical restrictions related to patient confidentiality.

## References

[1] Practice Committee of the American Society for Reproductive Medicine , “Evaluation and Treatment of Recurrent Pregnancy Loss: A Committee Opinion,” Fertility and Sterility 114, no. 6 (2020): 1151–1157. 10.1016/j.fertnstert.2012.06.048 22835448

[2] G. F. Grimbizis , S. Gordts , A. Di Spiezio Sardo , et al., “The ESHRE/ESGE Consensus on the Classification of Female Genital Tract Congenital Anomalies,” Human Reproduction 28, no. 8 (2013): 2032–2044. 10.1093/humrep/det098 PMC3712660 23771171

[3] R. Salim , B. Woelfer , M. Backos , L. Regan , and D. Jurkovic , “Reproducibility of Three‐Dimensional Ultrasound Diagnosis of Congenital Uterine Anomalies,” Ultrasound in Obstetrics & Gynecology 21, no. 6 (2003): 578–582. 10.1002/uog.127 12808675

[4] R. F. Valle and G. E. Ekpo , “Hysteroscopic Metroplasty for the Septate Uterus: Review and Meta‐Analysis,” Journal of Minimally Invasive Gynecology 20, no. 1 (2013): 22–42. 10.1016/j.jmig.2012.09.010 23312243

[5] C. Bermejo , P. Martínez Ten , R. Cantarero , et al., “Three‐Dimensional Ultrasound in the Diagnosis of Müllerian Duct Anomalies and Concordance With Magnetic Resonance Imaging,” Ultrasound in Obstetrics & Gynecology 35, no. 5 (2010): 593–601. 10.1002/uog.7551 20052665

[6] H. A. Homer , T. C. Li , and I. D. Cooke , “The Septate Uterus: A Review of Management and Reproductive Outcome,” Fertility and Sterility 73, no. 1 (2000): 1–14. 10.1016/s0015-0282(99)00480-x 10632403

[7] C. A. Venetis , S. P. Papadopoulos , R. Campo , S. Gordts , B. C. Tarlatzis , and G. F. Grimbizis , “Clinical Implications of Congenital Uterine Anomalies: A Meta‐Analysis of Comparative Studies,” Reproductive Biomedicine Online 29, no. 6 (2014): 665–683. 10.1016/j.rbmo.2014.09.006 25444500

[8] T. D. Deutch and A. Z. Abuhamad , “The Role of Three‐Dimensional Ultrasonography and Magnetic Resonance Imaging in the Diagnosis of Müllerian Duct Anomalies,” Journal of Ultrasound in Medicine 27, no. 3 (2008): 413–423. 10.7863/jum.2008.27.3.413 18314520

[9] J. F. W. Rikken , K. W. J. Verhorstert , M. H. Emanuel , et al., “Septum Resection Versus Expectant Management in Women With a Septate Uterus: An International Multicentre Open‐Label Randomized Controlled Trial (TRUST),” New England Journal of Medicine 385 (2021): 124–133. 10.1093/humrep/deab037 PMC8058590 33793794

